# Multiple immune-related adverse events secondary to checkpoint inhibitor therapy in patients with advanced cancer: association with treatment effectiveness

**DOI:** 10.3389/fonc.2024.1399171

**Published:** 2024-06-26

**Authors:** Cecilia Olsson Ladjevardi, Anthoula Koliadi, Viktoria Rydén, Ali Inan El-Naggar, Evangelos Digkas, Antonios Valachis, Gustav J. Ullenhag

**Affiliations:** ^1^ Department of Immunology, Genetics, and Pathology, Uppsala University, Uppsala, Sweden; ^2^ Department of Oncology, Uppsala University Hospital, Uppsala, Sweden; ^3^ Department of Oncology, Faculty of Medicine and Health, Örebro University, Örebro, Sweden; ^4^ Department of Oncology, Mälarsjukhuset, Eskilstuna, Sweden

**Keywords:** checkpoint inhibitors, multiple immune-related adverse events, immortal time bias, advanced cancer, cohort study

## Abstract

**Introduction:**

Checkpoint inhibitors (CPI) are widely used in cancer treatment with a potential of causing immune-related adverse events (IRAEs). Several studies have reported a positive correlation between development of IRAEs and improved survival outcome. However, few studies have focused on the potential role of multiple IRAEs on treatment effectiveness. This study aimed at investigating the association between multiple IRAEs and treatment effectiveness in terms of progression-free survival (PFS) and overall survival (OS) in advanced cancer patients.

**Methods:**

We performed a retrospective cohort study at three Swedish centers. All patients (n=600) treated with PD-L1 or PD-1 inhibitor, in monotherapy or in combination for advanced cancer between January 2017 and December 2021 were included. Multiple IRAEs were defined as IRAEs involving more than one organ system either simultaneously or sequentially. Time-depending Cox-regression model to mitigate the risk for immortal time bias (ITB) was applied.

**Results:**

The major tumor types were non-small cell lung cancer (205 patients; 34.2%) and malignant melanoma (196 patients; 32.7%). Of all patients,32.8% developed single IRAE and 16.2% multiple IRAEs. Patients with multiple IRAEs showed significantly improved PFS (Hazard Ratio, HR=0.78 95% Confidence Interval, CI: 0.57–0.98) and OS (HR=0.65 95% CI: 0.44–0.95) compared to patients with single IRAE or no IRAE (HR=0.46 95% CI:0.34–0.62 for PFS vs HR=0.41 95% CI: 0.28-0.60 for OS).

**Conclusion:**

In conclusion, our data supports a stronger association between development of multiple as opposed to single IRAEs and clinical effectiveness in advanced cancer patients treated with CPIs.

## Introduction

Immunotherapy with checkpoint inhibitors (CPI), i.e. anti-PD-1, anti PD-L1 and CTLA-4 antibodies has dramatically improved survival rates in various cancer types during the last decade and they are frequently used in different treatment settings (1–3). Although the introduction of CPIs has improved outcomes in several cancer types, their use cause a considerable risk for immune-related adverse events (IRAEs) which may appear in nearly every organ system and at any point during treatment and even after treatment discontinuation (4). Potential mechanisms resulting in IRAEs include increased T-cell activity against antigens present in both tumors and healthy tissue, increased levels of cytokines, and preexisting autoantibodies, and enhanced complement-mediated inflammation (4). Despite lack of knowledge considering the exact underlying pathophysiological mechanisms, the occurrence of IRAEs reflects activation of the immune system (4, 5). Since the development of IRAEs depends on the mechanism of action of CPIs, it has been assumed that patients developing IRAEs might have a better response to treatment.

The potential association between development of IRAEs and survival outcome is extensively studied. Several studies have shown a positive association between development of IRAEs and clinical benefit for different cancer types including malignant melanoma (MM), non-small cell lung cancer (NSCLC), renal cell carcinoma, urothelial cancer, head and neck cancer, and gastrointestinal cancer (6–10). Immune-related adverse events involving more than one organ, called multiple IRAEs have not been studied to the same extent. The current evidence suggests a better prognosis in patients with multiple IRAEs with a stronger effect magnitude compared to patients with single IRAEs (11–16), even if conflicting results exists (17). However, the current evidence can be questioned due to the high risk for immortal-time bias (ITB) that either has not been considered in some of the studies (14, 15) or it was dealt with in landmark analysis (12, 13, 17) that can also lead to bias compared to the more robust time-dependent Cox model (18). Besides, most of the studies only included patients with NSCLC (11–13, 16, 17), thus impacting the generalizability of study results.

The aim of the present study was to investigate the patterns of multiple IRAE occurrence and their impact on CPI effectiveness in an unselected cohort of patients with advanced cancer using time-dependent Cox models to mitigate the ITB risk.

## Patients and methods

### Study design and setting

In this multicenter retrospective cohort study, we identified all patients treated with CPIs (PD-1 or PD-L1 inhibitors) for advanced solid tumors between January 1^st^ 2017 until December 31^st^ 2021 from three regions (Södermland county, Uppsala county, Örebro county) in Sweden. Patients treated with a combination of PD-1 and anti-CTLA4 inhibitor were identified from local electronic prescribing systems for oncological therapy, alongside patients treated with CPIs as part of a clinical trial, and all were included in the analyses. We excluded patients treated with CPIs in a curative setting.

The study was approved by the Swedish Ethical Review Authority (reference number 019–02469 and 020–06801) and the requirement for informed consent was waived. The study has been performed in accordance with the principles of the Declaration of Helsinki.

### Data collection

Data were extracted from electronic medical records (EMR) by researchers (clinical oncologists) in a database with pre-specified variables of interest. The following data were collected: age at diagnosis (as years), sex, comorbidities expressed as Charlson Comorbidity Index, type of cancer, primary treatment at diagnosis, age at diagnosis of advanced cancer, metastatic sites, CPI initiation date, type of CPI, performance status (PS; WHO classification) at CPI initiation, number of previous lines of treatment, best treatment response on CPI, date of disease progression, IRAEs (date, type, grade, outcome), date of death and cause of death. IRAEs were collected before each treatment cycle or as acute events according to clinical practice.

### Outcomes and definitions

Immune-related adverse events were categorized in grade according to CTCAE 5.0 grading system. If the grade was not included in the EMRs, an approximation of the grade was decided based on the description of adverse events in EMRs and the laboratory findings, whenever feasible.

Multiple IRAEs were defined as IRAEs involving more than one organ system either simultaneously or sequentially.

Progression-free survival (PFS) was defined as the time from initiation of treatment to the occurrence of disease progression (as stated in the EMRs) or death. Overall survival (OS) was defined as the time from treatment initiation to death, irrespective of cause of death.

### Statistical methods

For descriptive statistics, numbers with percentages and median with range or interquartile range (IQR) were used for categorical and continuous variables, respectively. For bivariate analyses, either chi-square or Kruskal-Wallis test were used for comparisons among the different groups (no IRAE, single IRAE, or multiple IRAEs).

To identify factors associated with occurrence of IRAEs, logistic regression models were applied (no IRAE vs. multiple IRAEs or single IRAE vs. multiple IRAEs) to calculate Odds Ratios (OR) and their corresponding 95% Confidence Intervals (CI) using the following parameters as potential risk factors; age, sex, CCI, type of cancer, performance status, type of CPI, and treatment line.

To investigate the potential impact of IRAEs on time-to-event outcomes (PFS and OS), we performed time-dependent Cox regression models as the main analyses to calculate Hazard Ratios (HR) and their corresponding 95% CIs. Occurrence of the IRAE was considered as a time varying covariate. The rest of the covariates included were age, sex, CCI, performance status, type of CPI, type of cancer, and treatment line. A sensitivity analysis was performed by excluding all patients treated with combined CPI (monotherapy-only cohort). In addition, two subgroup analyses were performed based on cancer type (MM, NSCLC). Within NSCLC cohort, the analyses were stratified by treatment line.

All adjusted analyses were based on complete case approach, namely only cases with complete information for all the covariates included in each analysis were used.

Kaplan-Meier curves were used to visualize the impact of IRAEs on time-to-event outcomes. For the visualization of the distribution of organ systems involved in multiple IRAEs, a chord diagram was constructed.

All reported p-values were two-tailed with a 0.05 cut-off for statistical significance. All analyses were performed using SPSS (IBM Corp. Released 2021. IBM SPSS Statistics for Windows, Version 28.0. Armonk, NY: IBM Corp).

## Results

### Characteristics of study cohort

Baseline characteristics of the study cohort are summarized in **Table 1**. In total, 600 patients were included in the study cohort with a median age of 66 years (range: 21 – 87). The most common underlying malignant disease was NSCLC (205 patients; 34.2%) followed by MM (196 patients; 32.7%) and renal cell carcinoma (87 patients; 14.6%). Monotherapy with nivolumab was the most used CPI treatment (283 patients; 47.2%) followed by single treatment with pembrolizumab (211 patients; 35.2%) whereas 41 patients (6.8%) were treated with the combination of nivolumab and ipilimumab. Furthermore, 57 patients (9.5%) were treated with atezolizumab, 4 patients (0.7%) with durvalumab and 4 patients (0.7%) with cemiplimab. Median follow-up time for the overall cohort was 15 months (IQR: 6 to 28 months), 23 months (IQR: 13 to 40 months) for patients with PS of 0, 12 months (IQR: 6 to 24 months) for patients with PS of 1, and 7 months (IQR: 1 to 16.5 months) for patients with PS of 2.

**Table 1:** Characteristics of study cohort based on the occurrence of IRAE.

Variable	Whole cohort (N = 600) n (%)	No IRAE (N = 306) n (%)	Single IRAE (N = 197) n (%)	Multiple IRAEs (N = 97) n (%)	p-value
Age, median (range), in years	66 (21 – 87)	66 (21 – 87)	67 (24 – 87)	63 (24 – 84)	0.684
Sex Female Male	252 (42.0) 348 (58.0)	126 (41.2) 180 (58.8)	86 (43.7) 111 (56.3)	40 (41.2) 57 (58.8)	0.848
Charlson comorbidity index, median (range)	3 (0 – 11)	3 (0 – 11)	3 (0 – 9)	3 (0 – 11)	0.541
Type of cancer NSCLC Melanoma Renal cell carcinoma Urothelial carcinoma HNSCC Other	205 (34.2) 196 (32.7) 87 (14.5) 35 (5.8) 23 (3.8) 54 (9.0)	118 (38.6) 87 (28.4) 42 (13.7) 25 (8.2) 13 (4.2) 21 (6.9)	64 (32.5) 65 (33.0) 28 (14.2) 6 (3.0) 9 (4.6) 25 (12.7)	23 (23.7) 44 (45.4) 17 (17.5) 4 (4.1) 1 (1.0) 8 (8.2)	0.005
*de novo* metastatic disease	281 (46.9)	149 (48.7)	90 (45.7)	42 (43.3)	0.538
Visceral metastases	401 (66.8)	205 (67.0)	136 (69.0)	60 (61.9)	0.468
Central nervous system metastases	50 (8.3)	29 (9.5)	16 (8.1)	5 (5.2)	0.403
Performance status according to ECOG 0 1 ≥ 2	213 (35.7) 279 (46.7) 105 (17.6)	79 (26.0) 153 (50.3) 72 (23.7)	81 (41.1) 90 (45.7) 26 (13.2)	53 (55.2) 36 (37.5) 7 (7.3)	< 0.001
Type of checkpoint inhibitors Nivolumab Pembrolizumab Atezolizumab Nivolumab + Ipilimumab Durvalumab Cemiplimab	283 (47.2) 211 (35.2) 57 (9.5) 41 (6.8) 4 (0.7) 4 (0.7)	139 (45.4) 116 (37.9) 38 (12.4) 9 (2.9) 2 (0.7) 2 (0.7)	103 (52.3) 63 (32.0) 15 (7.6) 14 (7.1) 1 (0.5) 1 (0.5)	41 (42.3) 32 (32.0) 4 (4.1) 18 (18.6) 1 (1.0) 1 (1.0)	< 0.001
Line of treatment for checkpoint inhibitors 1^st^ 2^nd^ 3^rd^ or later	268 (45.0) 231 (38.8) 97 (16.3)	124 (40.5) 124 (40.5) 58 (19.0)	88 (44.7) 82 (41.6) 27 (13.7)	56 (57.7) 28 (28.9) 13 (13.4)	0.031

### Occurrence patterns, outcomes, and risk factors for multiple IRAEs

In total, 97 patients (16.2%) developed multiple IRAEs whereas 197 (32.8%) had a single IRAE during follow-up. Severity, management, and outcome of patients with IRAEs based on number of IRAEs is shown in **Table 2**. Patients with multiple IRAEs were more likely to develop grade ≥ 2 or grade ≥3 IRAEs that could lead to higher discontinuation rate compared to patients with single IRAEs. Patients with a single IRAE recovered without sequelae to a higher extent than patients with multiple IRAEs (60% vs. 44.3% p=0.027).

**Table 2:** Severity, management, and outcome of patients with immune-related adverse events (IRAEs) based on number of IRAEs.

	Single IRAE (N = 197) n (%)	Multiple IRAEs (N = 97) n (%)	p-value
Time to onset of IRAE(months), median (range)	2 (0 – 36)	1 (0 – 29)	0.925
Maximum grade of IRAE severity Grade ≥ 2 Grade ≥ 3	128 (65.0) 62 (31.5)	91 (93.8) 42 (43.3)	**< 0.001** **0.046**
Therapeutic management of IRAE* No treatment or supportive Corticosteroids Corticosteroids + alternative immunosuppression	82 (52.2) 69 (43.9) 6 (3.8)	40 (44.0) 45 (49.5) 6 (6.6)	0.351
Outcome of IRAEs Resolved without sequelae Resolved with minor sequelae Resolve with major sequelae	118 (60.0) 61 (31.0) 14 (7.0)	43 (44.3) 45 (46.4) 8 (8.3)	**0.027**
Death due to IRAE	4 (2.0)	1 (1.0)	0.533
Discontinuation due to IRAE	57 (28.9)	43 (44.3)	**0.009**

*Lack of information in 40 patients with single IRAE and 6 patients with multiple IRAEs.

Statistically significant results are presented in bold.

Risk factors for developing multiple IRAEs are shown in **Table 3**. Patients with PS ≥ 2 were less likely to develop multiple IRAEs (OR: 0.42 95% CI: 1.83–11.79) whereas combined CPI treatment was associated with development of multiple IRAEs (OR: 4.64 95% CI: 1.83–11.79) compared to no IRAE. We could not identify any risk factor associated with multiple IRAEs when compared to patients who developed a single IRAE.

**Table 3:** Risk factors for developing multiple immune-related adverse events.

Risk factors	Compared to no IRAE	Compared to single IRAE
	Odds ratio (95% Confidence Interval)	Odds ratio (95% Confidence Interval)
Age	0.99 (0.97 – 1.03)	0.99 (0.96 – 1.02)
Sex Female Male	1.13 (0.67 – 1.91) 1	0.96 (0.56 – 1.63) 1
Charlson comorbidity index	1.05 (0.87 – 1.27)	1.05 (0.85 – 1.31)
Type of cancer NSCLC Melanoma Other	0.87 (0.42 – 1.82) 1 0.92 (0.47 – 1.80)	0.91 (0.42 – 1.97) 1 0.84 (0.41 – 1.70)
Performance status according to ECOG 0 1 ≥2	1 0.62 (0.34 – 1.14) **0.42 (0.24 – 0.75)**	1 0.79 (0.44 – 1.44) 0.54 (0.21 – 1.40)
Type of checkpoint inhibitors anti-PD-1 anti-PD-L1 combination with anti-CTLA4	1 0.52 (0.18 – 1.47) **4.64 (1.83 – 11.79)**	1 0.89 (0.29 – 2.74) 2.18 (0.96 – 4.95)
Line of treatment for checkpoint inhibitors 1^st^ 2^nd^ 3^rd^ or later	1 0.79 (0.42 – 1.47) 0.78 (0.35 – 1.75)	1 0.66 (0.35 – 1.23) 0.94 (0.38 – 2.36)

Statistically significant results are presented in bold.

**Figure 1:**
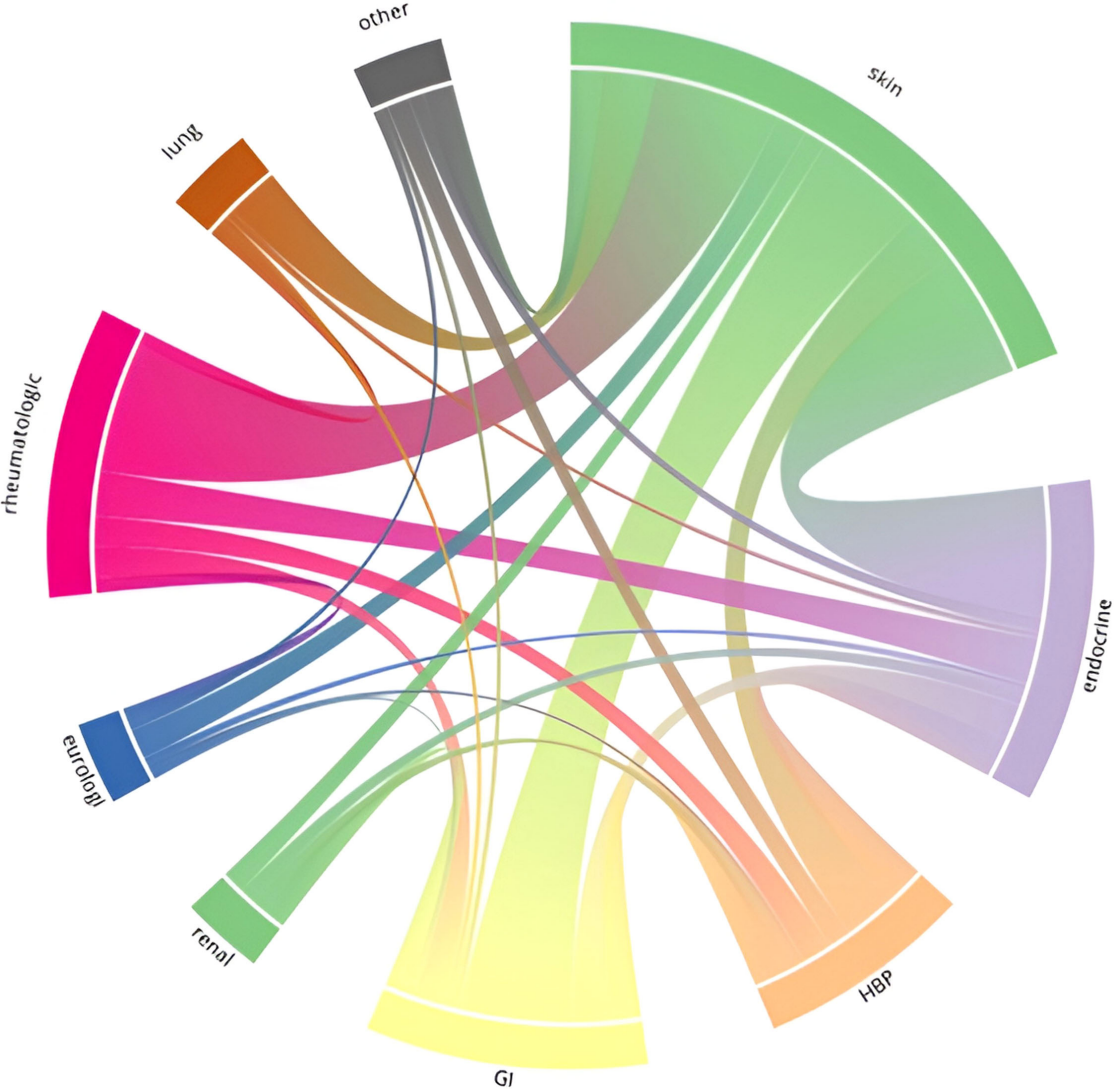
Chord diagram on the distribution of organ systems involved in multiple immune-related adverse events in a cohort of cancer patients with advanced disease treated with checkpoint inhibitors (n=600).

Distribution of organ systems involved in multiple IRAEs is demonstrated in **Figure 1**. The most common dyads of organs with IRAE development within the same patient were skin-gastrointestinal, skin-rheumatologic, skin-endocrine, and rheumatologic-endocrine.

### Impact of multiple IRAEs on PFS and OS

A summary of results from time-dependent Cox analyses regarding the occurrence of IRAE and prognosis in terms of PFS and OS is presented in **Table 4**.

**Table 4:** Impact of multiple IRAEs on disease prognosis according to time-depending Cox regression models.

Models*	Variables	Hazard Ratio (95% Confidence Interval)
Progression-free survival		
Time-dependent Cox, whole cohort (main analysis)	No IRAE Single IRAE Multiple IRAEs	1 0.78 (0.57 – 1.06) **0.46 (0.34 – 0.62)**
Time-dependent Cox, monotherapy only (sensitivity analysis)	No IRAE Single IRAE Multiple IRAEs	1 0.82 (0.59 – 1.16) **0.46 (0.34 – 0.63)**
Time-dependent Cox, NSCLC only (subgroup analysis)	No IRAE Single IRAE Multiple IRAEs	1 0.90 (0.52 – 1.56) 0.67 (0.39 – 1.15)
Time-dependent Cox, melanoma only (subgroup analysis)	No IRAE Single IRAE Multiple IRAEs	1 0.67 (0.39 – 1.12) **0.36 (0.22 – 0.59)**
Overall survival		
Time-dependent Cox, whole cohort (main analysis)	No IRAE Single IRAE Multiple IRAEs	1 **0.63 (0.43 – 0.92)** **0.41 (0.28 – 0.60)**
Time-dependent Cox, monotherapy only (sensitivity analysis)	No IRAE Single IRAE Multiple IRAEs	1 0.76 (0.52 – 1.13) **0.47 (0.32 – 0.68)**
Time-dependent Cox, NSCLC only (subgroup analysis)	No IRAE Single IRAE Multiple IRAEs	1 0.85 (0.45 – 1.60) 0.86 (0.47 – 1.59)
Time-dependent Cox, melanoma only (subgroup analysis)	No IRAE Single IRAE Multiple IRAEs	1 **0.46 (0.25 – 0.90)** **0.26 (0.14 – 0.50)**

*all analyses were adjusted for the following variables: age, gender, Charlson Comorbidity Index (CCI), performance status, type of checkpoint inhibitor, type of cancer (except from subgroup analyses), line of CPI treatment.

Statistically significant results are presented in bold.

**Figure 2:**
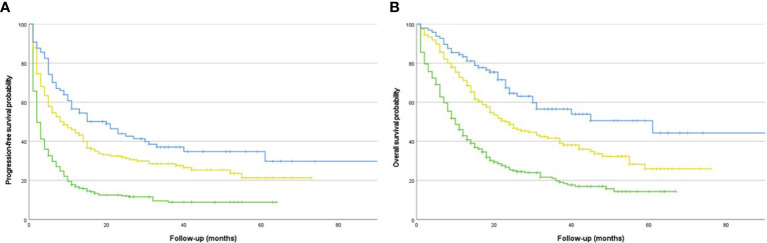
**(A)** Progression-free survival in a cohort of cancer patients with advanced disease treated with checkpoint inhibitors (n=600) based on the occurrence of immune-related adverse events. Blue line for multiple IRAEs; yellow line for single IRAE; green line for no IRAE. **(B)** Overall survival in a cohort of cancer patients with advanced disease treated with checkpoint inhibitors (n=600) based on the occurrence of immune-related adverse events. Blue line for multiple IRAEs; yellow line for single IRAE; green line for no IRAE.

The occurrence of multiple IRAEs was associated with statistically significant improvement in PFS (HR: 0.46; 95% CI: 0.34 – 0.63) compared to no IRAE in the whole study cohort as well as in the sensitivity analysis when only patients with CPI as monotherapy were included. An addition analysis with single IRAE as a reference demonstrated statistically significant improvement in PFS (HR: 0.78; 95% CI: 0.57–0.98) for patients developing multiple IRAEs compared to single IRAEs. A graphical visualization of PFS based on the occurrence or absence of IRAEs is shown in **Figure 2A**. In subgroup analyses, multiple IRAEs were associated with improved PFS in MM cohort but not in NSCLC cohort (**Table 4**).

In terms of OS, the occurrence of IRAEs resulted in statistically significant improvement in OS both in single IRAE (HR: 0.63; 95% CI: 0.43 - 0.92) and in multiple IRAEs (HR: 0.41; 95% CI: 0.28 - 0.60) cohorts (**Figure 2B**) compared to no IRAE. The association remained statistically significant in the sensitivity analysis with CPI monotherapy patients only for the occurrence of multiple IRAEs. In subgroup analyses, the occurrence of both single and multiple IRAEs was associated with improved OS in MM cohort whereas no similar association was observed in NSCLC cohort (**Table 3**). An addition analysis with single IRAE as a reference demonstrated statistically significant improvement in OS (HR: 0.65; 95% CI: 0.44–0.95) for patients developing multiple IRAEs compared to single IRAE.

After applying stratification based on treatment line in NSCLC cohort, a numerically lower HR for both PFS (n=77; HR: 0.51 95% CI: 0.21–1.23) and OS (HR: 0.58 95% CI: 0.20–1.67) for patients with multiple IRAEs when treatment was given as 1st line was observed compared to patients receiving CPI as 2^nd^ (n=137 PFS HR 0.85 95% CI: 0.39–1.87 OS HR 0.84 95% CI: 0.47–1.52) or later treatment line (n=57 PFS HR: 1.77 95% CI: 0.22–13.7 OS HR: 1.12 95% CI: 0.17–10.7).

In all main analyses, we could not reveal any difference in survival outcomes between PD-1 vs. PD-L1 treatment (HR for PFS: 0.84; 95% CI: 0.61 – 1.17; HR for OS: 0.94; 95% CI: 0.65 – 1.36). In an additional time-dependent Cox regression analysis of whether treatment discontinuation due to toxicity was associated with survival outcomes, we could not find a statistically significant association with either PFS (HR 0.78; 95% CI: 0.52 – 1.17) or OS (HR: 0.83; 95% CI: 0.53 – 1.29).

## Discussion

In our study cohort of 600 patients with advanced cancer treated with CPIs, we observed that approximately one-sixth developed multiple IRAEs. The occurrence of multiple IRAEs was associated with better treatment effectiveness as demonstrated by improvements in both PFS and OS with a magnitude of benefit significantly stronger compared to patients with single IRAEs.

Current evidence concerning development of multiple IRAEs for patients treated with CPIs and its impact on treatment effectiveness is scarce. There are only few previous studies (11–13, 16, 17), most of them indicating a stronger association between the development of multiple IRAEs, as opposed to single and survival (11, 12, 16). However, the cohorts in these studies included only patients with NSCLC and, therefore, the generalizability of study results can be questioned. Two earlier studies included patients with various malignant diseases, but smaller cohorts of approximately 200 patients (14, 15) demonstrated results in line with our larger cohort that provides more convincing evidence on this potential association in a broader patient population. A major methodological drawback of previous studies is how the risk for ITB was dealt. Immortal-time bias is a challenge to consider in the association between IRAEs and clinical outcome since patients responding to therapies continue treatment for a longer time and therefore increase their risk of developing IRAEs. To eliminate the risk of ITB in observational studies of survival outcomes established methodology such as landmark analysis, Cox model with time-varying variable or inverse-probability weighed models are routinely used (18–21). These methodological approaches cannot be considered as equal in terms of the validity of results since Cox model with time-varying variable seems to outperform landmark analysis (18). Considering the current evidence on potential association between multiple IRAEs and CPI effectiveness, some studies did not deal with ITB at all (14, 15) whereas others used landmark analysis only (12, 13, 17). We found two previous studies with a proper analysis of ITB reporting positive correlation between multiple IRAEs and outcome. However, both face issues related to generalizability of results to the clinical setting since only patients with NSCLC were included (11, 16) and one of the studies only analyzed patients participating in clinical trials and not in real-world setting (16). Until today, the current study is the first investigating the association between multiple IRAEs and CPI effectiveness in a broader cohort of patients with various malignancies using the preferable Cox regression model with time-dependent covariate as methodological approach for mitigating the ITB.

Subgroup analysis in our cohort showed statistically significantly improved PFS and OS for patients with MM but, as opposed to *Shankar et al*, not for patients with NSCLC. The lower number of NSCLC patients in our cohort compared to the study by Shankar et al. which only included NSCLC patients (n=205 vs. n=623) may, in part, explain this discrepancy (11). Interestingly, a trend towards improved PFS and OS for patients given CPI as a first line treatment that was diminished in later lines was observed in our cohort of patients with NSCLC highlighting treatment line as a source of heterogeneity among studies that could impact the results. This information was lacking from Shankar et al. and could also contribute to the discrepancy of study results. At the same time, one could argue that the ability of IRAEs to predict CPI effectiveness might depend on cancer type, a notion that is supported by the observations on the substantial differences on tumor immunogenicity among different cancer types (22, 23). In fact, the treatment effect is not equal in patients with different cancer types and differences in adverse effects among different cancer types have also been observed. A systematic review of 48 trials reported higher risk of developing skin and gastrointestinal IRAEs and lower risk of experiencing lung IRAEs for patients with MM compared to those with NSCLC whereas higher incidence of arthralgia and hypothyroidism in MM patients compared to patients with renal cell carcinoma was observed (5). Furthermore, it has been demonstrated that specific types of IRAEs exhibit a more profound correlation to treatment effectiveness and the type of these IRAEs differ across various cancer types. For example, vitiligo has been linked to treatment response in melanoma patients and thyroid dysfunction in NSCLC and renal cell carcinoma patients (24–28).

Checkpoint inhibitor combination therapy with PD-1 and anti-CTLA4 inhibitor is, for subgroups of patients, known to have better clinical efficacy than monotherapy, but the incidence of IRAEs is higher (29, 30). In our study cohort, we confirmed that the combination was a risk factor for development of multiple IRAEs. However, subgroup analyses of monotherapy only showed significantly better PFS and OS for patients developing multiple IRAEs, indicating that the improved outcome for patients with MM is not only due to the well-known better effect of combination therapy. Performance status ≥2 was associated with lower risk for developing multiple IRAEs in our study. A conceivable explanation is that patients with worse PS are more vulnerable in general with higher risk for earlier treatment discontinuation after the first IRAE episode and thus are less likely to develop multiple IRAEs.

Immunosenescent, a term describing impaired function of the immune system that develops with aging, has been supposed to influence the effect of immunotherapies and the development of IRAEs. However, many studies did not find a higher risk for development of IRAEs in older patients (31–34), but conflicting results exist (35). In a previous study from our research group, we found that a simplified frailty score based on PS, age and comorbidity expressed as Charlson Comorbidity Index (CCI) could predict development of all grade and multiple IRAEs, whereas age, CCI or PS did not separately predict increased risk for development of IRAEs (36). Although not supported by our adjusted analyses, another potential explanation of the association between PS and the risk of IRAEs might be more related to the line of treatment rather than the PS per se. In fact, some evidence suggests a negative impact of prior chemotherapy to immune microenvironment (37) that might influence the risk for IRAEs as well. In this equation, PS could serve as a surrogate for later treatment lines rather than as an explanatory parameter for the risk of IRAEs.

In terms of occurrence patterns and outcomes of IRAEs, we found a higher frequency of IRAE grade ≥ 2 and ≥ 3 in patients with multiple IRAEs than in patients with a single IRAE which could explain why a greater proportion of patients with multiple IRAEs than patients with a single IRAE discontinued treatment due to IRAE. At the same time, multiple IRAEs were not associated with higher risk for major sequelae from IRAEs. The organ systems involved in multiple IRAEs in this study consists of combinations of common single IRAEs without identifying any specific pattern. The number of patients within each pattern of distribution was too small and precludes any further analyses for potential associations of specific patterns with prognosis.

Besides from ITB potentially being the main bias in the association between IRAEs and clinical outcome, the present study has some additional limitations associated with its retrospective nature. These include the risk for misclassification bias regarding both IRAE grading but also classification between single and multiple IRAE as well as information bias where EMRs as data sources for identifying IRAEs might not include information on low grade IRAEs. The study is restricted to tree Swedish centers thus limiting its external validity whereas the relatively lower number of patients with tumor types other than melanoma and NSCLC limits the generalizability of the results. Finally, some variables of potential interest that could be associated with both the development of IRAEs and prognosis, as ethnicity, co-medication with immunosuppressive therapy, pre-existing autoimmune disease, were not available and were, therefore, not taken into account for the analyses. For some variables of interest as prior oncological treatment, the information was available, but the subgroups were too small for relevant analyses.

In conclusion, our study results suggest a statistically significant association between development of multiple IRAEs and CPI treatment effectiveness (measured as PFS and OS) that is mainly driven by patients with MM. These results support not discontinuing immunotherapy, even upon multiple but not severe IRAEs to increase the likelihood of treatment benefit. In addition, our findings suggest that multiple IRAEs may constitute a suitable surrogate marker for treatment efficacy that might be used in clinical trials. However, further studies with larger sample size and prospective design to overcome the inherent biases of retrospective studies is essential to further address the potential interplay between the development of IRAEs and treatment outcome.

## Data availability statement

The raw data are not available due to ethical restrictions but anonymized data might be available upon request.

## Ethics statement

The study was approved by the Swedish Ethical Review Authority (reference number 019-02469 and 020-06801). The studies were conducted in accordance with the local legislation and institutional requirements. Informed consent was waived from the Swedish Ethical Review Authority since the eligible patients were already treated with checkpoint inhibitors and the current study would not influence their subsequent treatment or prognosis.

## Author contributions

CO: Data curation, Writing – original draft. AK: Data curation, Writing – review & editing. VR: Data curation, Writing – review & editing. AI: Data curation, Writing – review & editing. ED: Data curation, Writing – review & editing. AV: Conceptualization, Formal analysis, Methodology, Project administration, Software, Supervision, Writing – original draft. GU: Conceptualization, Funding acquisition, Methodology, Supervision, Writing – original draft.
